# Socio‐economic status and time trends associated with early ART initiation following primary HIV infection in Montreal, Canada: 1996 to 2015

**DOI:** 10.1002/jia2.25034

**Published:** 2018-02-07

**Authors:** Vikram Mehraj, Joseph Cox, Bertrand Lebouché, Cecilia Costiniuk, Wei Cao, Taisheng Li, Rosalie Ponte, Réjean Thomas, Jason Szabo, Jean‐Guy Baril, Benoit Trottier, Pierre Côté, Roger LeBlanc, Julie Bruneau, Cécile Tremblay, Jean‐Pierre Routy, the Montreal Primary HIV‐Infection Study Group, L. Charest, C. Milne, S. Lavoie, J. Friedman, M. Duchastel, F. Villielm, F. Asselin, M. Boissonnault, P.J. Maziade, M. Milne, B. Lessard, M.A. Charron, S. Dufresne, M.E. Turgeon, S. Vezina, E. Huchet, J.P. Kerba, M. Poliquin, S. Poulin, P. Rochette, P. Junod, D. Longpré, R. Pilarski, E. Sasseville, L. Labrecque, C. Fortin, V. Hal‐Gagne, M. Munoz, B. Deligne, V. Martel‐Laferriere, M.E Goyer, N. Gilmore, M. Potter, M. Klein, M. Teltscher, A. de Pokomandy, L.P. Haraoui, Nathalie Rivet, Tuyen Nguyen, Nicole Bernard, Franck Dupuy, Eric A Cohen, Petronela Ancuta, Michel Roger, Mark A. Wainberg, Bluma G. Brenner

**Affiliations:** ^1^ Chronic Viral Illness Service McGill University Health Centre Montreal QC Canada; ^2^ Research Institute of the McGill University Health Centre Montreal QC Canada; ^3^ Department of Epidemiology, Biostatistics and Occupational Health McGill University Montreal QC Canada; ^4^ Department of Family Medicine McGill University Montreal QC Canada; ^5^ Department of Infectious Diseases Peking Union Medical College Hospital Beijing China; ^6^ Clinique Médicale l'Actuel Montréal QC Canada; ^7^ Clinique Médicale Quartier Latin Montréal QC Canada; ^8^ Clinique Médicale OPUS Montréal QC Canada; ^9^ Centre de recherche du Centre Hospitalier de l' Université de Montréal Montréal QC Canada; ^10^ Département de microbiologie infectiologie et immunologie Université de Montréal Montréal QC Canada; ^11^ Division of Hematology McGill University Health Centre Montreal QC Canada

**Keywords:** primary HIV infection, guidelines for the use of antiretroviral therapy, socio‐demographic factors, time trends, universal access to care, CD4 count, socio‐economic factors

## Abstract

**Introduction:**

Guidelines regarding antiretroviral therapy (ART) initiation in HIV infection have varied over time, with the 2015 World Health Organization recommendation suggesting ART initiation at the time of diagnosis regardless of CD4 T‐cell counts. Herein, we investigated the influence of socio‐demographic and clinical factors in addition to time trends on early ART initiation among participants of the Montreal Primary HIV Infection Study.

**Methods:**

The Montreal Primary HIV Infection Study is a prospective cohort established in three community medical centres (CMCs) and two university medical centres (UMCs). Recently diagnosed HIV‐infected adults were categorized as receiving early (vs. delayed) ART if ART was initiated within 180 days of the baseline visit. Associations between early ART initiation and socio‐demographic, socio‐economic and behavioural information were examined. Independent associations of factors linked with early ART initiation were determined using multivariable binary logistic regression analysis.

**Results:**

A total of 348 participants had a documented date of HIV acquisition of <180 days. The median interquartile range (IQR) age of participants was 35 (28; 42) years and the majority were male (96%), having paid employment (63%), men who have sex with men (MSM) (78%) and one to four sexual partners in the last three months (70%). Participants presented with a median IQR HIV plasma viral load of 4.6 (3.7; 5.3) log_10_ copies/ml, CD4 count of 510 (387; 660) cells/μl and were recruited in CMCs (52%) or UMCs (48%). Early ART initiation was observed in 47% of the participants and the trend followed a V‐shaped curve with peaks in 1996 to 1997 (89%) and 2013 to 2015 (88%) with a dip in 2007 to 2009 (22%). Multivariable analyses showed that having a paid employment adjusted odds ratio (aOR: 2.43; 95% CI: 1.19, 4.95), lower CD4 count (aOR per 50 cell increase: 0.93; 95% CI: 0.87, 0.99) and care at UMCs (aOR: 2.03; 95% CI: 1.06 to 3.90) were independently associated with early ART initiation.

**Conclusions:**

Early ART initiation during primary HIV infection was associated with diminished biological prognostic factors and calendar time mirroring evolution of treatment guidelines. In addition, socio‐economic factors such as having a paid employment, contribute to early ART initiation in the context of universal access to care in Canada.

## 1 Introduction

Human immunodeficiency virus (HIV) affects over 36.7 million people worldwide 1, 2. Nearly, half of those infected with HIV remain untreated, increasing their risks for acquired immune deficiency syndrome (AIDS), onward HIV transmission and mortality 3, 4, 5. While not a cure, antiretroviral therapy (ART) has substantially benefitted more than 18 million people infected with HIV for over two decades by controlling viral replication, AIDS and non‐AIDS events, and by reducing the risk of transmission 6, 7. However, the existence of latent viral reservoirs in CD4 T cells remains a hurdle to curing HIV infection and patients must remain on ART for the rest of their lives 2. We and others have reported that the predictors of HIV reservoir size include the nadir CD4 T cell count 8, CD4/CD8 ratio 9, the level of immune activation and the timing of initiation and duration of ART 10, 11, 12. In addition, early ART initiation has also been linked to normalization of CD8 T cell counts, further reducing the risk of non‐AIDS events 13.

Historically, decisions to initiate ART have been based on AIDS‐related clinical events, CD4 T‐cell count and comorbidities while trying to limit the risk of drug resistance and reduce ART‐related lipodystrophy and other toxicities 14, 15, 16, 17. Until 2008, treatment guidelines from the World Health Organization (WHO), Department of Health and Human Services (DHHS) USA, the European AIDS Clinical Society (EACS) and International AIDS Society (IAS) recommended ART initiation at CD4 count<350 cells/mm^3^ 6, 18, 19, 20. However, with the availability of more potent and tolerable ART, as well as data from the INSIGHT START and TEMPRANO studies 21, 22, early ART regardless of CD4 count is now uniformly recommended 1, 23, 24. CD4 count, therefore, is decreasingly considered a factor for treatment initiation and can be used as marker of a patient's immune and clinical status and prognosis 25.

ART initiation has also been linked to a variety of socio‐demographic and behavioural characteristics 26, 27, even in the context of publicly funded health care and medication access 28, 29, 30, 31. For instance, studies conducted in several African countries have shown that older age, female sex, pregnancy, lower psychological distress, no perceived communication barriers with providers and a gap in care of less than six months were associated with early ART initiation 26, 27. In 133 injecting drug users (IDUs) recruited in observational cohorts in Vancouver, Canada, Joseph *et al*. showed that recent calendar year and supervised methadone use were associated with early ART initiation whereby informal income generation and incarceration were associated with delayed ART initiation 28. Moreover, Cescon *et al*. observed that late ART initiation was more likely among females, non‐MSM and older age in 8942 participants in the Canadian Observational Cohort Study (CANOC) 29.

Understanding the various potential correlates of early ART initiation is important for informing ongoing and future care interventions. This understanding may also contribute towards achieving the Joint United Nations Program on HIV/AIDS (UNAIDS)'s ambitious goal for global ART programs, referred to as the 90‐90‐90 targets by the year 2020 32, 33. To this end, the Canadian statistics of 2014 report that an estimated 80% (73% to 87%) of persons living with HIV had been diagnosed, 76% (70% to 82%) of persons diagnosed with HIV were on treatment, and 89% (84% to 93%) of persons on treatment had suppressed viral load (VL) 34. Since increasing numbers of people will be offered ART over the long‐term, a better understanding of the factors playing a role in ART initiation may unveil potential barriers to overcome. Therefore, this study aimed to determine the role of socio‐demographic, socio‐economic, behavioural and clinical factors in early ART initiation during primary HIV infection (PHI) in Montreal, a setting where publicly funded universal health care and medication insurance are provided 35.

## 2 Methods

### 2.1 Study design and population

Established in 1996 at the time of onset of combination ART, the Montreal PHI Study 13, 36 is a cohort of early HIV‐1 infected individuals aged 18 years and above who are followed by physicians at either three community medical centres (CMCs) or at two university‐affiliated medical centres (UMCs). This flexibility allows participation of people who inject drugs (PWID) as universal access to care is provided by the Canadian healthcare system. Participants with an estimated date of HIV acquisition of less than 180 days were included and followed initially every three months for 24 months, with follow‐up being extended to 48 months in 2008. HIV diagnosis and ascertainment of PHI was performed using the guidelines proposed by the DHHS‐NIH Acute HIV Infection and Early Diagnosis Research Program 24 as described previously 37. Briefly, acute HIV‐1 infection (AHI) is defined as negative HIV p24 antibody testing by enzyme‐linked immunosorbent assay (ELISA) in the presence of detectable HIV‐1 RNA or positive HIV p24 antibody testing by ELISA and an evolving (≤3 bands positive) HIV Western blot. Early HIV‐1 infection (EHI) is defined by documented HIV‐1 acquisition within the previous six months. The HIV diagnosis and date of HIV acquisition was ascertained by a physician specialized in HIV at the participating study sites. The term PHI used in this study refers to both AHI and EHI. A structured case report form (CRF) was used to record the participants' socio‐demographic, socio‐economic, behavioural and clinical characteristics at first/baseline visit and each follow‐up visit.

ART initiation guidelines devised by national or international expert panels like that of DHHS, IAS, WHO and EACS in asymptomatic HIV‐infected adults varied during the study period as recently reviewed by Eholie *et al*. 38. Briefly, in late 1990s, the guidelines in the absence of evidence from randomized clinical trials (RCTs), recommended to start treatment at CD4 count of ≤500/mm^3^, which then dropped to CD4 count of ≤200 at the beginning of the 2000s owing to the growing concerns of long‐term drug toxicity. With the availability of data from early RCTs in 2006, this threshold was set at CD4 count of ≤350/mm^3^ for a brief period to reduce the higher risk of severe morbidity. This threshold was than increased to CD4 count of ≤500/mm^3^ by most panels during 2009 and 2013 and finally treatment for all patients was recommended since 2015.

### 2.2 Early versus late ART

The initiation of ART during follow‐up was recorded during subsequent visits at a maximum interval of three months. This binary outcome was defined as initiation of ART within 180 days of the first/baseline visit.

### 2.3 Socio‐demographic, socio‐economic and behavioural factors

The baseline socio‐demographic factors evaluated for their association with early ART initiation included age (years), sex (male vs. female), born in Canada (yes vs. no), ethnic background (Caucasian vs. non‐Caucasians) and mode of living (living alone vs. as a couple, with family and others). Socio‐economic factors included source of revenue (paid employment vs. on social welfare or unemployment benefits), personal annual income in Canadian dollars (below 10,000, 10,000 to 29,999, 30,000 to 49,999 vs. 50,000 and above) and more than elementary education (yes vs. no). Information on behavioural factors included exposure group (heterosexual, PWID, MSM vs. dual exposure group: that is, heterosexual and PWID) and number of sexual partners in past three months (zero, one to four vs. more than or equal to five partners).

### 2.4 Clinical factors

Testing for HIV‐1 p24 antigen and testing for HIV‐1 antibodies by EIA were performed in laboratories of the participating university‐affiliated medical centres. Confirmatory testing by Western blot was performed for all samples at the Laboratoire de Santé Publique du Québec. The ARCHITECT HIV Ag/Ab Combo assay was used for concurrent testing of p24 antigen and HIV‐1 antibodies after 2011 (Abbott Laboratories, Abbott Park, IL, USA). Laboratory testing also included measurements of plasma VL, CD4 and CD8 counts. Plasma VL was measured using the Roche Amplicor HIV Monitor assay (Roche Diagnostics, Mississauga, Ontario, Canada) until 2010 or the Abbott RealTime HIV‐1 assay (Abbott Laboratories) onwards.

### 2.5 Statistical analyses

Descriptive analyses were performed with means and standard deviations calculated for the variables with normal distribution and the median with interquartile range (IQR) calculated for variables with non‐normal distribution. Inferential analyses were conducted to measure the differences in socio‐demographic, socio‐economic, behavioural and clinical characteristics in association with early ART initiation at a level of significance of 5%, using bivariate logistic regression. The year of enrolment was also categorized into three‐year intervals (with the exception of the comparison group of 1996 to 1997 with a two‐year interval), which provided a robust sample size of over 35 participants in each category for further analysis. In addition, such an analysis strategy could also mitigate the potential influence of data artefacts. Mantel‐Haenszel χ^2^ test of trend was conducted for the analysis of ART initiation over years. Multivariable binary logistic regression analysis was conducted to determine the independent association of factors with early ART initiation. Using an exploratory analysis approach, all clinically relevant factors regardless of their statistical significance were simultaneously included in the multivariable model; no model reduction was performed. For two covariates that were highly correlated with each other (CD4/CD8 with CD4 and CD8; income with employment status) only one was selected; this decision was informed by consideration of missing data and clinical relevance. As the multivariable model does not consider cases with missing information, about 13.2% of such cases were only included in the univariable analysis. Interactions between year and other independent factors were assessed and were found to be insignificant (*p* > 0.05). Box‐Tidwell test was performed to check the assumption of linearity for quantitative variables in the multivariable model. The Hosmer‐Lemeshow goodness of fit test was performed to assess the fitness of the final model, which showed a reasonable fit (*p* = 0.741). Statistical significance in the univariable and multivariable analyses was defined as a two‐tailed *p* < 0.05. SPSS 23.0 (SPSS Inc., Chicago, IL, USA) and GraphPad Prism 7.0 (GraphPad Software, Inc. La Jolla, CA, USA) were used to perform and report on the statistical analyses.

### 2.6 Ethical considerations

The Montreal PHI Study was approved by the research ethics boards of all participating sites. The study was conducted in accordance with declaration of Helsinki and all study subjects provided written informed consent for their participation in the study.

## 3 Results

### 3.1 Characteristics of study participants

**Figure 1:**
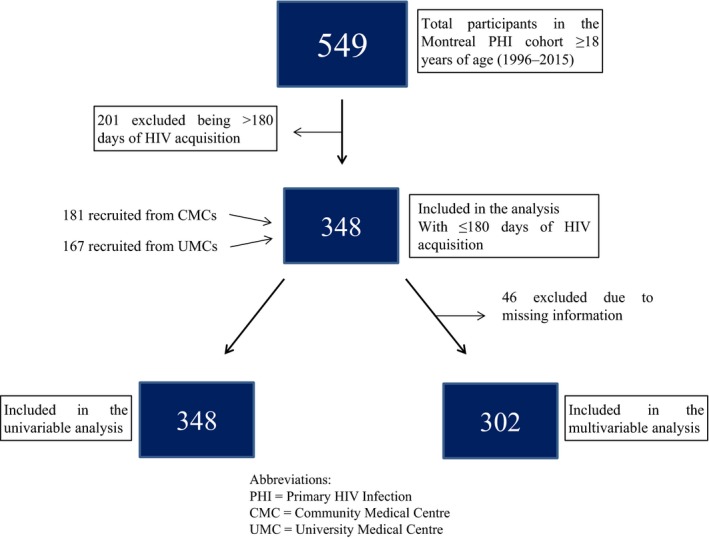
Flowchart describing study population distribution.

A total of 549 participants were recruited from 1996 to 2015, of which 348 (63%) were analysed as they had a documented date of HIV acquisition of <180 days with adequate follow‐up (Figure 1). The majority of participants were male (96%) with a median IQR age of 35 (28; 42) years at baseline. Furthermore, the vast majority of the study participants reported being born in Canada (81%), living alone (49%) and being Caucasian (89%). Among socio‐economic factors, a majority reported having paid employment (63%), an annual income of less than or equal to 30,000 Canadian dollars (56%) and more than elementary school education (91%). MSM was the most common exposure group (78%) and a majority of participants (70%) reported one to four sexual partners in the last three months. The baseline clinical presentation included median CD4 count of 510 (387; 660) cells/μl, CD8 count of 829 (600; 1232) cells/μl, CD4/CD8 ratio of 0.6 (0.4 to 0.9) and VL of 4.6 (3.7; 5.3) log_10_ copies/ml (Table 1).

**Table 1:** Baseline socio‐demographic, socio‐econonmic, behavioural and clinical characteristics of the study participants according to timing of ART initiation, (1996 to 2015)

Characteristics	Total (n = 348)	Early ART initiation		*p*	Bivariate OR (95% CI)^a^	Missing obs.
		Yes (n = 164)	No (n = 184)			
Socio‐demographic						
Age in years^b^	35.0 (28.0, 42.0)	37.0 (30.0, 43.0)	34.0 (27.0, 42.0)	0.015*	1.14 (1.03, 1.27)	0
Male	334 (96.0)	155 (94.5)	179 (97.3)	0.198	0.48 (0.16, 1.47)	0
Born in Canada	265 (81.0)	120 (73.2)	145 (78.8)	0.480	1.23 (0.70, 2.15)	21
Caucasian	311 (89.4)	141 (85.9)	170 (92.4)	0.056	0.51 (0.25, 1.01)	0
Mode of living						18
Alone	163 (49.4)	80 (48.8)	83 (45.1)	Ref.	Ref.	
Couple	83 (25.2)	38 (23.2)	45 (24.5)	0.625	0.88 (0.52, 1.49)	
Family	32 (9.7)	13 (7.9)	19 (10.3)	0.383	0.71 (0.33, 1.53)	
Others	52 (15.8)	24 (14.6)	28 (15.2)	0.713	0.89 (0.48, 1.67)	
Socio‐economic						
Paid employment	208 (62.7)	104 (63.4)	104 (56.5)	0.155	1.39 (0.88, 2.17)	16
Annual income						38
Below 10,000$	79 (25.5)	37 (22.6)	41 (22.3)	Ref.	Ref.	
10,000 to 29,999$	93 (30.0)	43 (26.2)	49 (26.6)	0.937	0.98 (0.54, 1.78)	
30,000 to 49,999$	94 (30.3)	47 (28.7)	47 (25.5)	0.678	1.14 (0.62, 2.07)	
Above 50,000	44 (14.2)	17 (10.4)	27 (14.7)	0.381	0.72 (0.34, 1.51)	21
More than elementary education	299 (91.4)	141 (86.0)	158 (85.9)	0.663	1.19 (0.54, 2.60)	
Behavioural						
Exposure group						0
Heterosexual	27 (7.8)	18 (11.0)	9 (4.9)	Ref.	Ref.	
PWID	27 (7.8)	16 (9.8)	11 (6.0)	0.574	0.73 (0.24, 2.20)	
MSM	270 (77.6)	122 (74.4)	148 (80.4)	0.038*	0.41 (0.18, 0.95)	
Dual	24 (6.9)	8 (4.9)	16 (8.7)	0.020*	0.25 (0.08, 0.80)	
Multiple partners						13
Zero partners	28 (8.4)	14 (8.5)	14 (7.6)	Ref.	Ref.	
One to four partners	234 (69.9)	110 (67.1)	124 (67.4)	0.765	0.89 (0.41, 1.94)	
≥5 partners	73 (21.8)	33 (20.1)	40 (21.7)	0.666	0.83 (0.35, 1.97)	
Clinical						
CD4 count (cells/μl)^c^	510 (387, 660)	440 (333, 611)	540 (447, 683)	<0.001***	0.90 (0.86, 0.95)	5
CD8 count (cells/μl)^d^	829 (600, 1232)	876 (589, 1276)	810 (615, 1207)	0.329	1.01 (0.99, 1.02)	6
CD4/CD8 ratio	0.59 (0.38, 0.86)	0.51 (0.32, 0.82)	0.64 (0.46, 0.91)	0.255	0.77 (0.49, 1.21)	6
VL, log_10_ copies/ml	4.6 (3.7, 5.3)	4.8 (3.7, 5.6)	4.4 (3.7, 5.0)	0.002**	1.36 (1.12, 1.64)	2
Recruitment centre						
CMCs	181 (52.0)	76 (46.4)	105 (57.1)	Ref.	Ref.	
UMC	167 (48.0)	88 (53.7)	79 (42.9)	0.046*	1.54 (1.01, 2.35)	0

Values are median (Q1, Q3) or number of participants (%). ART, antiretroviral therapy; Ref., reference category; obs., observations; others, friends or institution; PWID, people who inject drugs; MSM, men who have sex with men; VL, viral load; UMC, university medical centre; CMC, community medical centre

^a^OR, odds ratio, 95% CI confidence interval estimated from bivariate logistic regression analyses.

^b^Age OR and 95% CI calculated per five year increase.

^c^CD4 OR and 95% CI calculated per 50 cells/μl increase.

^d^CD8 OR and 95% CI calculated per 50 cells/μl increase.

*p < 0.05; **p < 0.01; ***p < 0.001.

### 3.2 Early ART initiation, its associated factors and trends over time

Early treatment, which was defined as starting within 180 days of baseline visit, was initiated in approximately half of the participants (n = 164, 47%). When compared to those who did not initiate early ART, bivariate analysis demonstrated that these participants were significantly older in age (OR per five‐year increase: 1.14; 95% CI: 1.03, 1.27) with no differences observed between males versus females, being born in Canada versus outside Canada, and living alone versus other living modes (living as a couple, a family, or with friends). Socio‐economic factors were not associated with early ART initiation. Compared to participants in the heterosexual exposure group, MSM (OR: 0.41; 95% CI: 0.18, 0.95) and those in the dual exposure group (OR: 0.25; 95% CI: 0.08, 0.80) were significantly less likely to initiate early ART. Other factors significantly associated with early ART initiation in the bivariate analyses were a lower CD4 count (OR per increase of 50 cells/μl: 0.90; 95% CI: 0.86, 0.95), a higher VL (OR per log_10_ copies/ml increase in VL: 1.36; 95% CI: 1.12, 1.64) and being recruited in UMCs versus CMCs (OR: 1.54; 95% CI: 1.01, 2.35) (Table 1). In addition, participants at CMCs versus UMCs presented with a higher median CD4 count (530 vs. 497 cells/μl; *p* = 0.044), which may explain the 10% difference in early ART initiation between these two medical clinics (Data not shown).

**Figure 2:**
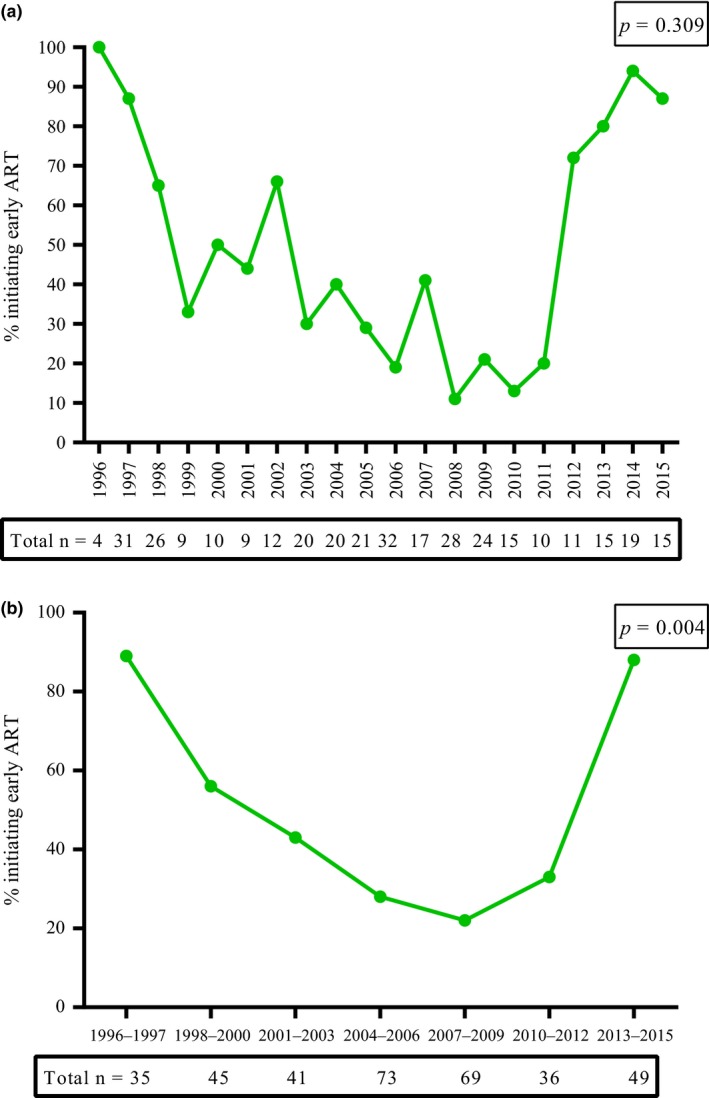
(a) Trends of early ART initiation over years during primary HIV infection in Montreal; 1996 to 2015. (b) Trends of early ART initiation over three‐year intervals during primary HIV infection in Montreal; 1996 to 2015. ART, antiretroviral therapy.

The majority of early cohort (1996 to 1998) and late cohort (2012 to 2015) participants initiated early treatment, with proportions reaching approximately 90% by the years 2014 to 2015 (Figure 2a). However, proportions of participants initiating ART early between 2003 and 2011 remained below 40% (Figure 2a). We further categorized the years into three‐year intervals (with the exception of the comparison group of 1996 to 1997 having a two‐year interval). Early ART initiation was significantly associated with calendar time grouped into such three‐year intervals (χ^2^ for trend *p* = 0.004). A V‐shaped curve of early ART initiation was observed when the years were categorized by such three‐year intervals (which provided a sample size of greater than 35 participants in each category for further analysis (Figure 2b)). Multivariable analysis showed that having paid employment versus being on social benefits/unemployment insurance adjusted odds ratio (aOR: 2.43; 95% CI: 1.19, 4.95), lower CD4 cells/μl (aOR per 50 cell increase: 0.93; 95% CI: 0.87, 0.99), greater CD8 cells/μl (aOR per 50 cells/μl increase: 1.02; 95% CI: 1.00 to 1.04) and receiving care at UMCs versus CMCs (aOR: 2.03; 95% CI: 1.06 to 3.90) were independently associated with early ART initiation.

When compared to the years 1996 to 1997, early ART initiation was significantly less likely during the years from 1998 to 2012 independent of other factors, while it was similar for the most recent years 2013 to 2015 (aOR: 0.39; 95% CI: 0.06 to 2.36) (Table 2). These findings highlight that early ART initiation is not only associated with evolving treatment recommendations (prognostic factors and calendar year), but also with socio‐economic characteristics such as employment.

**Table 2:** Multivariable analysis of factors associated with early ART initiation

Characteristics	aOR	95% CI	*p*
Socio‐demographic			
Age in years^a^	1.17	0.99, 1.39	0.055
Male	0.38	0.08, 1.96	0.249
Born in Canada	2.62	0.92, 7.44	0.071
Caucasian	0.44	0.12, 1.66	0.225
Mode of living			
Alone	Ref.	Ref.	Ref.
Couple	0.61	0.29, 1.29	0.198
Family	0.92	0.30, 2.80	0.885
Others	1.44	0.60, 3.44	0.417
Socio‐economic			
Paid employment	2.43	1.19, 4.95	0.015*
More than elementary education	0.98	0.29, 3.30	0.968
Behavioural			
Exposure group			
Heterosexual	Ref.	Ref.	Ref.
PWID	0.45	0.07, 2.76	0.389
MSM	0.60	0.18, 2.01	0.412
Dual	0.45	0.10, 2.07	0.307
Multiple partners			
Zero partners	Ref.	Ref.	Ref.
One to four partners	1.59	0.41, 6.24	0.504
≥5 partners	0.92	0.22, 3.80	0.906
Clinical			
CD4 count (cells/μl)^b^	0.93	0.87, 0.99	0.039*
CD8 count (cells/μl)^c^	1.02	1.00, 1.04	0.031*
VL, log_10_ copies/ml	1.21	0.89, 1.64	0.220
Recruitment centre			
CMCs	Ref.	Ref.	Ref.
UMC	2.03	1.06, 3.90	0.033*
Calendar year^d^			
1996 to 1997	Ref.	Ref.	Ref.
1998 to 2000	0.08	0.02, 0.37	0.001**
2001 to 2003	0.03	0.01, 0.13	<0.001***
2004 to 2006	0.02	0.01, 0.11	<0.001***
2007 to 2009	0.01	0.00, 0.05	<0.001***
2010 to 2012	0.03	0.01, 0.17	<0.001****
2013 to 2015	0.39	0.06, 2.36	0.302

aOR, adjusted odds ratio, 95% CI confidence interval estimated from multivariable logistic regression analysis. ART, antiretroviral therapy; Ref., reference category; others, friends or institution; PWID, people who inject drugs; MSM, men who have with men; VL, viral load; UMC, University Medical Centre; CMC, Community Medical Centre.

^a^Age OR and 95% CI calculated per five year increase.

^b^CD4 OR and 95% CI calculated per 50 cells/μl increase.

^c^CD8 OR and 95% CI calculated per 50 cells/μl increase.

^d^Calendar year categorized per three‐year interval with the exception of reference group (1996 to 1997).

*p < 0.05; **p < 0.01; ***p < 0.001.

## 4 Discussion

Early diagnosis and prompt treatment initiation are considered key factors in controlling HIV epidemic. Based on which, UNAIDS has adopted 90‐90‐90 target that by 2020 ≥90% of persons living with HIV infection (PLWH) will know their HIV status, ≥90% of PLWH will be on ART and ≥90% will achieve viral suppression on ART 32. Achieving these targets by 2020 would lead to an end of the AIDS epidemic by 2030. Considered a window of opportunity, ART initiated in PHI has been associated with decreased seeding of latent reservoirs, near normalization of immune activation, reduction in the risk for progression to AIDS and non‐AIDS illnesses and a substantial reduction in the risk for secondary transmission 7, 10, 11, 39, 40, 41. PHI therefore provides the best opportunity for early ART initiation. Our study determined associations between socio‐demographic, socio‐economic, behavioural, clinical characteristics and calendar year with early ART initiation in participants of the Montreal PHI study. Nearly half of the participants initiated early ART, which was independently associated with having paid employment, lower CD4 and elevated CD8 T‐cell counts, receiving care at a UMC and remote as well as more recent calendar years. Recent trends show over 90% of participants initiating early ART, which is encouraging given the clinical and population benefits inherent in currently recommended treatment guidelines by WHO, DHHS and Quebec ministry of health 1, 19, 23.

We observed a significant association between increasing age with early ART initiation in the bivariate analysis, which decreased to marginal significance when considering other covariates in the multivariable model. In contrast, Cescon *et al*. reported late ART initiation in older adult participants from the CANOC 29. This difference may be due to our analysis focusing on patients diagnosed during primary HIV infection and the differing study period, 1996 to 2015 versus 2000 to 2012 for the CANOC study. Our finding, however, is consistent with a large prospective study (Swiss HIV Cohort) where younger age was associated with delayed ART initiation 42. Of note, in both the CANOC and Swiss studies, early ART initiation was defined based on CD4 counts, whereas our study defined ART initiation as occurring within six months of the PHI diagnosis/the baseline visit. We observed an independent association of early ART initiation with lower CD4 counts, which is comparable with CANOC study findings and has been associated with faster disease progression.

Degroote *et al*. have reported on the associations of socio‐economic, behavioural and clinical factors with health‐related quality of life in HIV‐infected individuals in Belgium 43. They reported incapacity to work as an important determinant of patient outcomes highlighting the role of socio‐economic factors. Similarly, Kesselring *et al*. linked lower socio‐economic status with late ART initiation in 61 participants of the ENGAGE Cohort Study in British Columbia, Canada 44. These results are consistent with our observation of having a paid employment contributing to early ART initiation during PHI. We, however, did not include annual income in the multivariable model due to its significant association with employment status (χ^2^ *p* < 0.001 and Cramer's V = 0.697; *p* < 0.001; Data not shown). Employment is considered a component of economic status, and plays an important role in an individual's overall health and access to healthcare 45. Our findings indicate unemployment status as a potential barrier to care despite the context of universal care including ART medication where only a maximum copayment of CAN $87.16 per month is expected for those not on social welfare and are covered by the provincial insurance. Interestingly, we observed delayed ART initiation in those not having a paid employment and this group included those on social welfare. In similar settings such as Spain where no copayment is required, Perez‐Molina *et al*. also reported an association of lower socio‐economic status with late ART initiation 46. In the resource‐limited settings with no universal health coverage such as Uganda, improved employment and schooling outcomes were reported in households of HIV‐infected adults with high CD4 counts owing to early initiation of ART 47. Therefore, employment and socio‐economic status should be considered if a better control of HIV epidemic is expected. Inclusion of social support programs into the HIV care centres focusing on individuals with lower socio‐economic status and/or without employment may reduce such barriers to early ART initiation.

Boyer *et al*. recently described early ART initiation rates and associated factors in 514 HIV‐infected individuals enrolled in a universal test and treat cluster randomized trial in South Africa 48. They reported an early ART initiation rate of 88% from 2012 to 2015, which is consistent with our result for a similar time period. Similarly, in both studies lower CD4 T‐cell count was associated with early ART initiation. They also reported an association between early ART initiation and having a regular sexual partner. On the other hand, we asked about the number of sexual partners in the past three months, which did not show an association with early ART initiation. These differences may be due to the way the question was asked, and the predominant female participants in their South African study (70.4%) compared to a small proportion of our study participants (4.0%). In addition, we report time trends in early ART initiation resembling a V‐shaped curve over the years from 1996 to 2015. The trends in ART initiation reflect evolving treatment recommendations over time: “treat early and treat hard” in the mid‐1990s, changing to initiation only when CD4 < 200 cells/mm^3^, and the current practice of treating HIV irrespective of CD4 count 1, 23, 49, 50. Due to the recognition of long‐term toxicities of ART including lipodystrophy and the potential for the development of drug resistance, treatment guidelines recommended treatment in asymptomatic patients only until their CD4 counts declined to below 200 51, 52. With recent advancements in antiretroviral drug regimens with limited toxicity and higher efficacy, guidelines now recommend treatment initiation regardless of CD4 counts 1, 19.

ART initiation in this cohort was also independently associated with type of care centre; that is participants at UMC versus CMC were more likely to initiate ART within six months (aOR: 2.03; 95% CI: 1.06, 3.90). However, when ART initiation was defined as within one year of baseline visit, no difference between the two clinical settings was observed (Results not shown). This unexpected finding also led us to explore possible differences in participant characteristics in these two settings. Participants seeking care at the CMCs did not differ from those seeking care at the UMCs in terms of age and CD4:CD8 ratio (Results not shown). However, participants at UMCs versus CMCs presented with a lower median CD4 count (*p* = 0.044), which could have led these participants to initiate early ART. Such statistical differences between median CD4 counts in CMCs versus UMCs may not be clinically meaningful as very limited opportunistic infections or comorbidities may develop at a difference of 33 CD4 cells/μl). Another possible explanation is that patients who are acutely ill tend to go to the hospital emergency rooms, where referral is biased to the ambulatory follow‐up in hospitals. Therefore, symptomatic presentation may be important for understanding this difference. The presence of AIDS‐defining diseases as well as diagnoses such as TB, hepatitis B, hepatitis C, lymphoma and HIV‐associated chronic comorbidities such as renal disease may have influenced decisions to initiate ART. Therefore, missing information about these concomitant illnesses may have limited our capacity to examine the presence of these factors in the increased likelihood of early ART initiation in UMCs. Nonetheless, the frequency of the AIDS‐defining illness is extremely rare during the initial years of infection and the prevalence of TB, hepatitis C and hepatitis B are very low in men having sex with men in the province of Quebec. This difference in early ART initiation by type of centre, however, was independent of socio‐demographic factors. In addition, higher plasma VL and lower CD4 counts have previously been shown by our group to be associated with severe clinical presentation 53, 54. Another possible explanation requiring further insight is the comparatively more institutional nature of the UMCs with different practice patterns of physicians and/or more support services for patients. Similarly, there may have been less concern for lipodystrophic changes induced by ART 55 and its social image by the institutional physicians. In addition, patients might have been treated earlier at UMCs by participating in the clinical trials and possibly academic physicians were more likely to follow most recent research findings rather than therapeutic guidelines. Therefore, a better description of the clinical site attributes, medical provider training and treatment‐related knowledge and beliefs could help to explain this finding. Besides quantitative research, a qualitative approach to further explore the barriers to early treatment in the patients in these two types of centres can be useful.

Our study has several limitations which need to be considered while interpreting the results. First, our study population was predominantly comprised of MSM which limits the generalizability of study findings. The inclusion of only 14 female participants limited our analysis on the potential influence of gender on ART initiation in this study. However, the MSM group represents the majority (53%) of persons living with HIV in Canada despite the presence of small regional variations 56. Among 249 new HIV cases in Quebec during the year 2015, 76% were MSM 57. Furthermore, among the key HIV populations in Canada, the incidence of HIV has remained significantly higher and stable in the MSM group over the last decade. Therefore, such findings have important implications in Canada as well as for countries with similar HIV epidemics. Furthermore, it is unknown how representative participants in the Montreal study are of patients experiencing PHI. We have no information on non‐participation to help inform presence of a selection bias. Since, 63.4% of the total study participants (n = 549) contributed to the current analysis, we compared age, sex and income distribution of this group (n = 348) with the rest (n = 201) to evaluate potential selection bias. This analysis showed a similar age (*p* = 0.845), sex (*p* = 0.131) and income distribution (*p* = 0.099) among the two groups. Moreover, information was missing for some of the participants in clinical and socio‐demographic characteristics which did not allow for multivariable modelling. Similarly, information on the number of sexual partners in the last three months was asked as a close‐ended question with limited number of categories due to sensitivity of the information and to increase response. In addition, information on geographic factors was not collected due to ethical concerns, limiting our analysis regarding distance to care. Therefore, there could be residual confounding due to lacking/missing information and unavailability of clinically important variables such as mental health status. This study also did not include information on signs and symptoms which could be a confounding factor for the association between early ART initiation and type of care centre. The substantial fluctuation in the ART initiation rates over the years in our study may be influenced by data artefacts, which may have been mitigated by categorizing year into three‐year intervals. Finally, our sample size did not allow performing analyses stratified by type of care centre to assess the possible effect modification on socio‐economic status and early ART initiation. Such limitations need to be considered in future large scale studies.

Nevertheless, our study findings suggest the presence of a health inequity in access to healthcare even in the context of universal access to care. Besides overcoming the challenges in early diagnosis of HIV infection 58, early ART initiation represents an effective strategy in reducing morbidity and mortality among HIV‐infected persons 59, 60. Nearly a decade ago, our group was among the first proponents of ART initiation at early stages of infection based on our knowledge of local transmission dynamics and foreseeable benefits for the individual and community 5, 61, 62. With recent guidelines recommending treatment for all at the time of HIV diagnosis, monitoring HIV clinical cohorts for inequities in access to early ART would be important to further document the nature and persistence of this phenomenon. Of note, the Montreal PHI Study also represents a unique cohort, where participants treated early could also be invited to participate in future HIV eradication studies.

## 5 Conclusions

Globally, calendar year, prognostic factors and socio‐economic characteristics were associated with early ART initiation during primary HIV infection. An ongoing consideration of socio‐economic characteristics is important as interventions are developed to achieve the UNAIDS goals of 90‐90‐90 and ultimately curtail the HIV epidemic.

## Competing interests

The authors have no competing interests to declare.

## Authors' contributions

VM, JC and JPR conceived and designed the study. VM and JPR were involved in the data acquisition. VM, JC and JPR performed the statistical analyses and data interpretation. VM, JC and JPR drafted the manuscript. JPR also obtained funding and administrative support for the study. VM, JC, BL, CC, WC, TL, RP, RT, JS, JGB, BT, PC, RL, JB, CT and JPR contributed to editing and critical revision of the manuscript for important intellectual content. All authors read and approved the final manuscript.

## Appendix 1

### 1.1

We are thankful to the physicians and/or researchers from the Montreal Primary HIV infection study group of the participating centres for their collaboration: Drs L. Charest, C. Milne, S. Lavoie, J. Friedman, M. Duchastel, F. Villielm, F. Asselin, M. Boissonnault, P.J. Maziade, M. Milne at Clinique medicale l'Actuel, Montreal; Drs B. Lessard, M.A. Charron, S. Dufresne, M.E. Turgeon, S. Vezina, E. Huchet, J.P. Kerba, M. Poliquin, S. Poulin, P. Rochette, P. Junod, D. Longpré, R. Pilarski, E. Sasseville at Clinique medicale du Quartier Latin, Montreal; Drs L. Labrecque, C. Fortin, V. Hal‐Gagne, M. Munoz, B. Deligne, V. Martel‐Laferriere, M.E. Goyer at Centre Hospitalier de l'Université de Montréal, Montréal, QC, Canada, Pavillon Hotel‐Dieu and Notre‐Dame, Montreal; Drs N. Gilmore, M. Potter, M. Klein, M. Teltscher, A. de Pokomandy, L.P. Haraoui at Chronic Viral Illness Service, McGill University Health Centre, Montreal; Drs. Nathalie Rivet and Tuyen Nguyen at Hôpital de la Cité‐de‐la‐Santé, Laval, QC. We are obliged with the support extended to Montreal PHI Study by the researchers in the Réseau SIDA/Maladies infectieuses du FRQS: Dr. Nicole Bernard and Dr. Franck Dupuy, Infectious Diseases and Immunity in Global Health Program at the Research Institute of the McGill University Health Centre, Montreal; Dr. Eric A Cohen, Director of the Human Retrovirology Research Unit, Montreal Clinical Research Institute (IRCM), Montreal; Drs. Petronela Ancuta and Michel Roger, Centre de recherche du Centre Hospitalier de l'Université de Montréal, Montréal; Dr. Mark A. Wainberg and Dr. Bluma G. Brenner, McGill University AIDS Centre, Montreal, Canada.
